# Stakeholder Perspectives on Fertilizer Policy in Germany: Findings from a Modified Delphi Study

**DOI:** 10.1007/s00267-025-02266-3

**Published:** 2025-09-27

**Authors:** Jannik Aaron Dresemann

**Affiliations:** Thünen Institute of Farm Economics, Braunschweig, Germany

**Keywords:** Fertilizer Policy, Nutrient Balancing, Stakeholder Participation, Bottom-up Policy Demand, Environmental Management

## Abstract

The European Farm to Fork strategy mandates transformative measures to reduce agriculture’s environmental impacts, yet its translation into actionable policies remains ambiguous. In Germany, current fertilizer policies rely on rigid, action-oriented guidelines that stakeholders increasingly criticize for failing to address complex environmental challenges. This study captures policy demands for improved nutrient management by engaging primary stakeholders—from agriculture, environmental protection, and academia—through a modified policy Delphi process. Iterative online working groups and a plenary scenario workshop, structured around a systematic framework on environmental policy instrument selection, elicited open-ended, demand-driven responses. Findings reveal a strong consensus for shifting from prescriptive fertilization practices to target-oriented, scientifically grounded approaches. Stakeholders advocate adopting farm-gate nutrient balancing to reduce nitrogen losses and manage phosphorus surpluses while emphasizing the need for robust monitoring systems enhanced by digital technologies. This participatory approach integrates diverse expert perspectives into policy recommendation, enhancing the legitimacy and adaptability of future fertilizer policies while reducing political dissent. Although these stakeholder-driven recommendations offer promising directions for reconciling agricultural productivity with environmental sustainability, further empirical research—including pilot projects and simulation studies—is needed to validate feasibility and refine the methodological framework. The insights from this study contribute to the bottom-up development of fertilizer policy instruments that support the broader objectives of the Farm to Fork strategy.

## Introduction

Numerous studies have delineated the influence of current agricultural production systems on the environment and their vulnerability to climate change (Stoate et al., 2009; Bindi and Olesen 2011; Sánchez-Bayo and Wyckhuys 2019). In response to these challenges, the European Commission launched the Farm to Fork strategy as a central component of the European Green Deal, aiming to improve the sustainability of food systems, including the objective to reduce nutrient losses by at least 50% (European Commission 2020). This EU-wide initiative is complemented by national and regional policies targeting the environmental consequences of agriculture and its adaptation to climate pressures (Böcker and Finger 2016).

Recent research has assessed the potential impacts of such policies, including yield reductions, land-use shifts, and production relocation within and beyond the EU (Beckman et al., 2020; Barreiro-Hurle et al., 2021; Bremmer et al., 2021; Henning et al., 2021). In Germany, several studies have documented nutrient surpluses and their environmental consequences, emphasizing the urgency of improved nutrient management (Haenel et al., 2020; Löw and Osterburg 2024). Despite this, less attention has been paid to the actual demands of the key stakeholders, such as farmers, advisory bodies, and environmental groups who must comply with and implement these policies in practice.

Although the Farm to Fork strategy sets clear environmental objectives, it offers little guidance on the specific policy instruments required to achieve them. Even though fertilizer regulation has long been part of the EU’s environmental agenda (Council of the European Union 1991; European Parliament 2000, 2006), the complexity of designing effective and acceptable measures remained (BMEL 2023). In particular, there is a need to reconcile scientific, political, and stakeholder perspectives, especially where trade-offs between environmental and economic objectives arise.

Although there is scientific literature on various policy instruments that could be used to reduce nutrient losses, there likely is a gap between what politicians and stakeholders agree upon and the scientific view (Likens 2010). Therefore, referring solely to the scientific literature to determine the framework conditions seems to be insufficient. This study, therefore, shifts the focus from pure policy design to a demand-centered narrative. A more precise understanding of regulatory requirements necessitates the evaluation of a likely socially accepted regulatory framework that considers the needs of key stakeholders and is grounded in scientific evidence.

The objective of this study is to identify feasible and effective policy instruments for reducing nutrient losses in German agriculture, as envisioned by the Farm to Fork strategy. It seeks to answer the research question: *What new or modified policy instruments, or combinations thereof, are considered appropriate to motivate farmers to reorganize their production in such a way that the objectives of the Farm to Fork strategy for fertilization are achieved with limited public resources?*

To explore this, a modified policy Delphi process was applied. This method enabled iterative stakeholder engagement and structured discussions based on established criteria for environmental policy instrument choice (Scheele et al., 1992; Siebert 2008). The process was designed to elicit open-ended, demand-driven responses from stakeholders representing agriculture, environmental protection, and academia. The diversity of perspectives aimed to ensure both relevance to practice and alignment with scientific evidence.

This paper proceeds as follows: Section 2 ”State of the Art” reviews the state of knowledge on nutrient losses in Germany and stakeholder engagement in environmental policy design. Section 3 “Methods: Scenario Development Integrating Stakeholder Demand” presents the methodological framework. Section 4 “Results: Stakeholder Demand for Fertilizer Policies” outlines the findings of the Delphi process, which are then discussed in Section 5 “Discussion” in light of the method and the literature. The paper concludes with key implications for policy and future research directions.

## State of the Art

### Nutrient Losses in Germany

A growing body of research and monitoring data provides insights on nitrogen (N) and phosphorus (P) surpluses in German agriculture and their environmental consequences. These insights are critical for assessing the baseline conditions against which the nutrient reduction targets of the EU Farm to Fork Strategy must be evaluated.

Drawing on data from over 5900 farms in the German Farm Accountancy Data Network, Löw and Osterburg (2024) present a comprehensive analysis of N balances and nitrogen use efficiency (NUE) across different farm types. The study reveals substantial disparities: pig and poultry farms exhibit the highest N surpluses (156 kg N/ha) and the lowest NUE (49%), while arable farms perform significantly better, with surpluses of 18 kg N/ha and an NUE of 94%. Dairy farms and mixed systems fall in between, with dairy farms recording the lowest average NUE at 40%. The analysis also highlights the effects of external nutrient flows, particularly feed imports and manure exports, which can mask off-farm N losses when not properly accounted for in nutrient balances.

P surpluses are similarly problematic, especially in livestock-dense regions. The Federal Government (2021) emphasizes that manure-based fertilization often exceeds crop uptake, resulting in long-term P accumulation and increased risk of runoff. According to the authors, the findings highlight the need for spatially differentiated P management strategies to sustainably reduce P levels in heavily oversupplied soils. Additionally, improved monitoring of P flows is essential to address legacy surpluses in regions with high concentrations of organic fertilizer use.

Complementing the farm-level data, long-term spring water monitoring by Weber and Kubiniok (2022) in Saarland and Rhineland-Palatinate shows a strong correlation between nitrate concentrations in groundwater and the proportion of cropland in surrounding catchments. While pollution hotspots have improved due to targeted measures, overall nitrate levels have continued to rise in many locations. The authors call for stronger enforcement and tighter water protection regulations to mitigate diffuse agricultural pollution.

Recent empirical studies have evaluated the implications of regulatory N limits on agronomic and economic outcomes. Kage et al. (2022), analyzing fertilization responses in winter oilseed rape and wheat, found that existing thresholds under the German Fertilizer Ordinance (DüV) fall approximately 18–20% below economic optima. While this leads to improved N balances, it may reduce protein content in cereals and limit profitability in high-demand crops. Moreover, further reductions in nitrate-sensitive “red zones” are unlikely to substantially reduce leaching, due to already diminished marginal effects.

Contrasting these findings, Taube (2023) argues, based on modeled relationships between N surplus and nitrate leaching, that current N demand values should be lowered by 15–20% to meet the legal targets of the DüV. Taube contends that such reductions would enhance environmental outcomes, particularly in regions with historically high N loads, and would encourage broader adoption of best management practices.

Also, digital tools are frequently promoted as a means to improve site-specific fertilization. However, Heyl et al. (2023) caution that while digital precision technologies can enhance NUE and reduce losses, their effectiveness is constrained by governance limitations, including enforcement gaps, rebound effects, and weak integration into broader sustainability frameworks. The authors argue that quantity-based instruments such as livestock-to-land ratios or emissions trading are better suited to addressing the structural causes of nutrient surpluses.

Taken together, these findings emphasize the limitations of one-size-fits-all regulation. While the sectoral average N balance of 63 kg N/ha and NUE of 60% may appear acceptable, high variability across and within farm types necessitates more tailored approaches. Recent research highlights the need for differentiated strategies that account for farm structure, regional nutrient loads, and the varying efficiency potentials across systems (Löw and Osterburg 2024).

### Stakeholder Participation in Environmental Policy Design

Traditional models of public policy describe a linear progression—from problem definition through formulation, implementation, and evaluation (Anderson 1979; Lindblom 1980; Jones 1984; Portney 1986; Starling 1988; Dye 2002). However, fertilizer regulation in Germany highlights the complexity of environmental policymaking, which have led to protracted debates between agriculture and water protection in recent years (Schaub 2021). In 2016, the European Commission found Germany in breach of the EU Nitrates Directive for failing to take effective action against nitrate pollution. This resulted in a 2018 ruling by the European Court of Justice (European Court of Justice 2018), triggering legislative revision and a commitment to impact monitoring (BMEL 2023).

Against this backdrop, the Farm to Fork Strategy’s targets demand substantial improvements in NUE without compromising productivity, posing new challenges to policy makers. These challenges underline the need to understand stakeholder positions and design policy instruments that balance environmental objectives with sector-specific realities. A more differentiated and adaptive approach, while complex, may increase policy acceptance and effectiveness (Starling 1988; Altman 1994).

Furthermore, participatory approaches are increasingly recognized as essential for designing effective and legitimate environmental policies (Tippett et al., 2007; Reed 2008). Involving stakeholders early in the policy process can improve acceptance, reduce implementation barriers, and enhance the quality of decisions (Fiorino 1990; Chess and Purcell 1999). However, meaningful participation requires more than consultation, it must empower stakeholders to influence outcomes (Welp et al., 2006; Fischer and Young 2007).

Stakeholder integration also needs to ensure that policy alternatives are not only informed by diverse perspectives but, as far as possible, also are grounded in scientific evidence. A substantial body of research has identified the necessity of scientific information and analysis in providing stakeholders with relevant information to make informed decisions. Therefore, incorporation of academia is considered to be a crucial component within participatory processes (Webler et al., 1991; Chess et al., 1998; Chase et al., 2004; Fischer and Young 2007; Tippett et al., 2007; Reed 2008).

One structured method to operationalize such stakeholder engagement in complex and contested policy fields is the policy Delphi method. This approach is a structured, iterative approach designed to explore divergent views and identify areas of consensus and dissent among experts and stakeholders (Turoff 1970; Linstone and Turoff 1975). Unlike the traditional Delphi approach, which aims for consensus among a group of experts, the policy Delphi method seeks to incorporate diverse and strongly opposing views regarding potential solutions to significant policy issues (Linstone and Turoff 1975; Belton et al., 2019).

Policy issues of this kind typically lack definitive experts and instead involve informed advocates and referees. While an expert or analyst can provide quantifiable or analytical estimates of the effects resulting from a particular policy resolution, it is improbable that such an analysis will lead to a universally accepted resolution of the policy issue; if it did, the issue no longer would be a matter of policy. In the policy Delphi context, the individual experts assume the role of advocates for effectiveness or efficiency and must contend with advocates representing various interest groups within the relevant society or organization (Turoff 1970). In this study, which relates to a highly controversial political topic (Schaub 2021), the latter applies in particular to the experts from agricultural and environmental interest organizations, while the experts from academia are regarded as referees. Recent applications of Delphi methods in agricultural and environmental contexts have demonstrated its value in capturing stakeholder preferences and informing policy design (Belton et al., 2019; Ehlers et al., 2022). However, methodological transparency—especially regarding expert selection, iteration design, and data analysis—is critical to ensure credibility and replicability (Rintamäki et al., 2016; Devaney and Henchion 2018).

## Methods: Scenario Development Integrating Stakeholder Demand

Environmental policy-making increasingly requires the integration of diverse stakeholder perspectives to ensure legitimacy, feasibility, and effectiveness. This study adopts a participatory approach grounded in best-practice principles (Reed 2008), aiming to capture stakeholder-supported policy preferences for nutrient management in Germany. The process was designed to empower participants from agriculture, environmental protection, and academia to actively shape the discussion on future fertilizer policy instruments.

Unlike traditional top-down policy design, this study employed a modified policy Delphi method to facilitate structured, iterative dialog among stakeholders. The process was not limited to consultation but enabled participants to influence the framing of policy options through a series of online workshops and a final plenary scenario workshop. These sessions were guided by a predefined framework for environmental policy instrument selection (Scheele et al., 1992), which structured the discussion around five key parameters: costs, technological starting point, addressees, regulatory areas, and instruments.

By incorporating stakeholder perspectives into the development of fertilizer policy instruments, this study aims to generate policy recommendations that are both scientifically sound and socially accepted, thereby advancing the Farm to Fork Strategy’s objective of reducing nutrient losses. Early and structured stakeholder engagement serves as a foundation for formulating clear, targeted policy demands that reflect the practical realities and normative expectations of key actors. The following sections present the methodological framework that guided the study, detailing the criteria for selecting environmental policy instruments, the study design, and the procedures for expert sampling and workshop implementation.

### Criteria for the Generation of Environmental Policy Measures

Policy design entails the intentional and strategic effort to define policy objectives and align them with instruments or measures capable of achieving the desired outcomes (Howlett et al., 2015). Hence, effective environmental policy design requires an in-depth understanding of the nature of environmental challenges and a structured approach to instrument selection. This is in particular true for the advancement of fertilizer policies in the light of the Farm to Fork strategy.

Foundational studies by Baumol and Oates (1971), Dewees (1983), Keohane et al. (1998), Richards (1998), and Goulder and Parry (2008) explore the economic, political, and administrative dynamics involved in choosing regulatory tools for environmental management. Scheele et al. (1992) in turn, provide a practically oriented decision-making framework, delineating five critical parameters for the design of environmental policy measures: costs, technological starting point, addressees, regulatory areas, and policy instruments. This approach is particularly valuable when addressing complex agricultural and environmental systems, as it links strategic policy decisions directly to sector-specific technological and economic realities. Its continued relevance is supported by more recent scholarship emphasizing the importance of structured, criteria-based approaches to policy formulation (Howlett et al., 2015; Jordan and Turnpenny 2015). By providing a structured framework for policy development, the model proposed by Scheele et al. (1992) not only enriches broader theoretical discourse but also offers practical guidance for applied policy-making, thereby justifying its adoption in this study.

According to Richards (1998) an efficiency-seeking government will reduce the sum of three cost components: production cost (direct cost of pollution abatement), implementation cost, and public finance cost for policy instrument selection. The choice of policy alternatives therefore is influenced by variations in transaction cost components, which themselves are shaped by the instrument selected (Richards 1998; McCann 2013) In alignment with this framework, the present study adopts the cost typology proposed by Scheele et al. (1992): opportunity costs, arising from the foregone benefits of alternative production activities; administrative costs, incurred through the implementation of measures; control costs, associated with monitoring and enforcement; and consensus-building costs, which reflect the political and institutional efforts required to achieve stakeholder agreement.

In order to overcome environmental problems regarding nutrient management, emissions must be reduced. However, emissions cannot always be clearly and cost-effectively traced back to their sources. Therefore, proxy variables frequently are employed as the technological starting point for regulatory initiatives when selecting policy measures (Siebert 2008). In the area of fertilization, these proxies can, for example, be based on mineral fertilizer sales or the farm nutrient balance.

The aim of a measure should be to encourage emitters and users to change their behavior. However, farmers may not be the only addressees of a measure. Legislators can pass costs forward or back among different actors along the supply chain and select not only farmers but also retailers or consumers as addressees. The extent to which these actors will pass on the impetus of a measure to farmers depends on the nature of the problem and the technological starting point.

The need to identify an adequate regulatory area is driven by the fact that environmental problems have a spatially differentiated structure and regulations should relate to the diffusion characteristics and sources of pollution. Reduction in the size of regulatory areas has the effect of increasing the costs associated with administration and control (Scheele et al., 1992).

Finally, the type and level of regulatory impetus to tackle an environmental problem depend on the choice of instrument, which can be combined with different starting points, addressees, and regulatory areas. In general, a distinction must be made between two basic types: quantity-controlling instruments and price-controlling instruments. Quantity-controlling instruments (requirements, licenses) are able to address the volume of emissions directly. A requirement provides each issuer with a fixed framework for action, such as conditions on timing and amount of organic fertilizer application, while a license allows the implementation of the requirements to be transferred between issuers. Price-controlling instruments (levy, subsidy) send price signals that are intended to bring about a reduction in emissions through economically oriented action. The difference between a levy and a subsidy is that the interest in reducing emissions is based on increasing revenue in the case of a subsidy and on reducing costs in the case of a levy, e.g., per kilogram N in mineral fertilizer.

In addition, policymakers may apply mixed forms when combining different decision parameters. It is important to note that there may be interdependencies between the individual parameters when assessing mixed forms. This is particularly relevant in the context of the technological starting point, the addressee, and the control area. For instance, if the emitter is not addressed, a product (input, output) must be selected as the technological starting point. This also predetermines the choice of a regulatory area in which the product can be traded at the same conditions. Mixed forms therefore are classified as small-scale differentiable if the issuer is addressed directly and as non-differentiable if measures address companies upstream or downstream in the supply chain.

### Modified Delphi Study—Multi-stakeholder approach

**Fig. 1:**
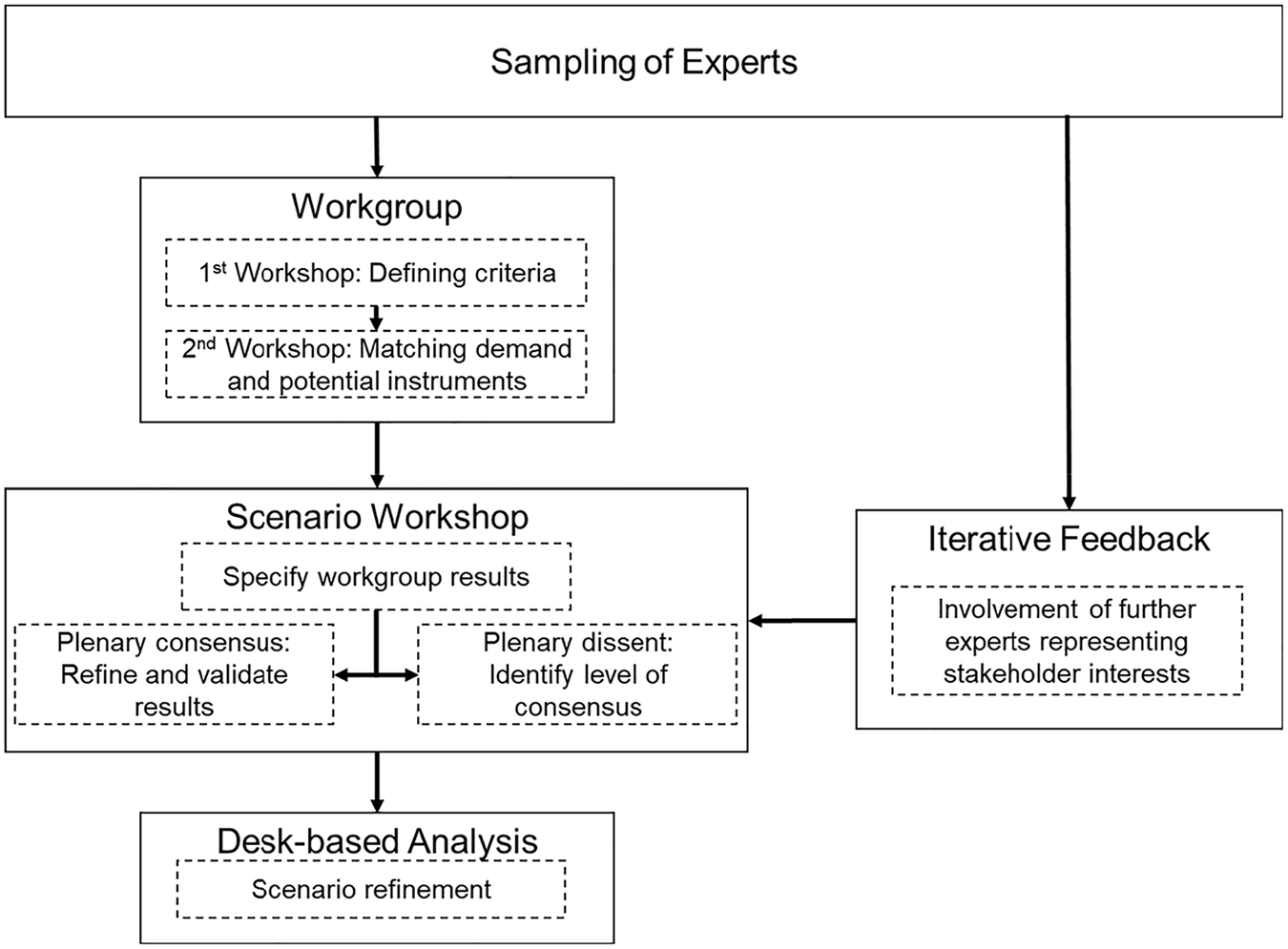
Process of Scenario Development

In this study, the policy Delphi setting was operationalized through an iterative series of online workshops with stakeholders, culminating in a final online plenary scenario workshop that included additional stakeholder representatives (Fig. 1). To foster deliberation and gain insight into the rationale behind policy preferences, the process began with the distribution of invitations in June 2023, followed by a reminder four weeks later. The first thematic working group session was held in September 2023, and the second in November. The final synthesis and scenario workshop, which brought in a broader set of experts not involved in the earlier working group sessions, took place in February 2024. Unlike conventional survey-based approaches, this Delphi process enabled discourse-oriented, iterative engagement, allowing participants to articulate their positions, negotiate trade-offs, and co-develop policy preferences grounded in both practical experience and scientific evidence (Reed 2008; Belton et al., 2019; Ehlers et al., 2022). This approach aligns with recent calls for more participatory and adaptive policy design processes that reflect the complexity of land-use systems and the diversity of actor perspectives (Oberlack et al., 2023).

#### Expert Sampling

In a policy Delphi study, expert selection is pivotal to the robustness and credibility of the findings (Turoff 1970). The applied quota sampling approach prioritized expertise, transparency, and balance (Rintamäki et al., 2016; Devaney and Henchion 2018). It sought a balance between shared goals and divergent perspectives, grounded in the institutional profiles of the relevant organizations, to stimulate meaningful exchange during scenario development (Krueger and Casey 2014). Therefore, experts were recruited from three relevant stakeholder domains in German nutrient governance: agriculture, environmental protection, and academia to reflect important positions and interests surrounding fertilizer policy.

To foster intensive and constructive deliberation, the number of participants in the final scenario workshop was limited to twelve. This manageable group size was chosen to enable in-depth discussion while maintaining sufficient heterogeneity for meaningful scenario exploration. To promote candid exchange and reduce social desirability bias, participants remained anonymous in all external reporting. While participants were known to one another during the sessions and engaged in direct interaction, it was agreed that individual contributions would not be attributed by name in any publications.

Participants were selected based on clear and transparent criteria (Rowe and Wright 2001; Caley et al., 2014). For stakeholder representatives, selection was guided by the relevance of their institutions within the German policy landscape and their individual experience. Key selection criteria included: years of experience, membership in advisory committees, peer nominations, perceived standing within the expert community, and the strategic relevance of their stakeholder group (McBride and Burgman 2012).

For academic participants, relevant institutions were first identified, and individuals were selected based on multiple criteria: advanced academic qualifications, a strong publication record related to agriculture or environmental protection, and active involvement in policy debate. All selected academic experts met these criteria, ensuring that the scientific perspective in the study was grounded in both academic competence and policy engagement.

Prior to participation, all experts received a project brief detailing the study’s background, objectives, and research questions. This ensured informed consent and alignment with the study’s scope. The composition of the expert panel involved in the process is outlined in Table 1, which provides an overview of gender, seniority, and disciplinary background. While the stakeholder categories were broadly grouped into agriculture, environmental protection, and academia, disciplinary backgrounds in agri-environmental governance were covered across all groups.

**Table 1:** Attributes of Participants

Stakeholder Group	Female	Male	Senior	Junior	Disciplinary Backgrounds
Agriculture		4	4		Agronomy, agri-environmental policy, farm management
Environmental protection^a^	1	2	2	1	Biodiversity conservation, agri-environmental policy, nutrient-cycling
Academia^a^		5	5		Biodiversity and sustainable land use, governance, agri-environmental policy

^a^The scenario workshop and one workgroup meeting were not attended by one participant from the group

Although the number of participants in this study is limited to twelve, the panel composition prioritized depth, relevance, and institutional representation over demographic breadth. The majority of participants held senior positions within key stakeholder organizations, including national-level farming associations, environmental NGOs, and public agencies. While their positions provided them with a broad overview of sectoral developments and stakeholder perspectives, all participants contributed to the study in their personal capacity as experts. This approach allowed for a more open and reflective exchange, while still ensuring that the insights gathered were grounded in extensive professional experience and informed by close engagement with relevant policy and practice contexts. As such, the sample, though small, offers a high degree of relevance and analytical depth for understanding the dynamics of nutrient governance at the national level (McBride and Burgman 2012).

#### Workgroup Process for Generating and Validating Policy Measures

Prior to the initial working group session, the core of scientific, production-related, and legal knowledge on fertilization, as well as the state of the political science discussion on the design of efficient agri-environmental policies, was compiled by the author and made available to the participants. This preparatory literature research facilitated a meaningful discussion, including technical aspects such as the selection criteria for environmental policy measures (Richards et al., 2004; Reed 2008). On this basis, the working group of six experts discussed and developed the key points of the policy demand, which were then validated and extended by an additional group of experts representing the involved stakeholder groups as well as academia during the scenario workshop.

As the working group meetings built on each other, there was an iteration of the previous decision-making process after both workshops. Workgroup decisions were documented in the minutes of the meeting and subsequently served as the foundation for the subsequent meeting agenda. Furthermore, all the action parameters that were deliberated and endorsed in the working group meetings were also reviewed and ratified in plenary during the scenario workshop. The total number of iterations was three, with the instruments being incorporated into only the second and third iteration.

In order to facilitate the process and reduce coordination effort, the workgroup meetings as well as the plenary scenario workshop were conducted online via Microsoft Teams, including Microsoft Whiteboard, and were moderated by the author. All participants received an agenda as well as key questions in advance of the meetings to guide the discussion. Audio recordings of the discussion rounds were transcribed and subsequently used to provide the participants with a written protocol and to serve as the basis for the analysis. The open-ended questions that guided the discussion were developed based on the findings of the literature review and systematically divided into the different problem dimensions based on the decision parameters from Scheele et al. (1992) (Appendix A). Conflicts that emerged during the discussions—particularly concerning the use of different N performance indicators—were addressed by the author and subsequently summarized in written form for the participants. This supplementary information was based on additional literature research and consultations with external scientific experts who had contributed to relevant publications (Reed 2008). At the end, consensus and dissent between the stakeholders were recorded if no compromises were reached among the participants.

During the initial workgroup meeting, participants were invited to share their perspectives as representatives of their respective stakeholder groups, fostering a shared understanding of the policy context and framing the discussion. To structure stakeholder input, a common time horizon extending to 2030 was established, and the European Union was defined as the maximum geographical scope for potential regulatory measures. Building on this foundation, the overarching environmental objective of reducing nutrient losses by 50% was critically examined in light of existing national legislation. This policy objective was then systematically deconstructed into its core components using the decision-making framework proposed by Scheele et al. (1992), which is summarized in Chapter 3.1.

To move from individual perspectives to a unified group opinion, the initial meeting split the fertilization workgroup into two subgroups. Each subgroup discussed the suitability of the current national policy instruments for achieving the objective. Based on this, each subgroup established the requirements for the action parameters, regulatory scope, technological starting point, and addressees. The results of the subgroups were subsequently presented to each other for collective agreement on the parameters’ design. In the second workshop, experts examined potential policy instruments emerging from these decision parameters, using a “lowest common denominator” as a baseline for discussion. Both consensus and dissent were recorded during this stage, informing the plenary scenario workshop. The final scenario workshop, scheduled two months in advance in alignment with participant availability, aimed to broaden the range of perspectives and validate the previously selected decision parameters and policy instruments by including six additional external experts. However, due to scheduling conflicts, one of the newly invited experts and one member of the initial fertilization working group were unable to attend, resulting in a total of ten expert participants.

To avoid bias toward any group, the coordinator maintained balanced representation across the three stakeholder groups in both the working group sessions and the plenary scenario workshop. This procedure was guided by three key objectives: first, to gain a comprehensive understanding of stakeholder demands by engaging a diverse range of experts; second, to validate preliminary results through broader expert feedback; and third, to design a process that is both time- and cost-efficient while still accommodating extensive stakeholder input.

The final scenario workshop on stakeholder demand and corresponding political implementation options was conducted analogous to the working group meetings: It was held online and moderated by the author. In advance, all participants received a report on the course of the discussion and the results from the working group to generate a uniform level of knowledge on interim results. The report was intended to guide the discussion in the workshop and to focus on areas of dissent. The intention was to encourage participants to position themselves with regard to these controversies. Prior to the plenary scenario workshop, working hypotheses were formulated by the author based on the discussion of core components and areas of dissent in the workgroup. These were further elaborated on and adapted by experts, who then negotiated the characteristics until a consensus or dissent was reached on specific action parameters in the plenary scenario workshop. The results were summarized in a report, which was distributed among the experts to be checked for plausibility and consistency with the workshop discussion.

## Results: Stakeholder Demand for Fertilizer Policies

The results for policy design to reduce fertilizer losses, as presented below, were achieved during the plenary scenario workshop under the assumption that the objective of the Farm to Fork strategy to reduce nutrient losses by 50% is implemented at the European level and that the interventions are not a matter of a solo national effort. The structure of the result sections is based on the criteria for the selection of environmental policy measures, as outlined in Chapter 3.1. Unless otherwise specified, consensus is defined as unanimous agreement (100%) among all participating experts during the final discussions in plenary.

### Technological Starting Point

During the first workgroup meeting, two technological starting points were discussed: mineral fertilizer and nutrient losses. One argument was that if mineral fertilizers were selected as a starting point, the competitiveness of organic fertilizers could be enhanced by increases in the price of mineral fertilizers. The economic value of nutrients contained in organic fertilizers would rise and, as a result, organic fertilizers would be used more efficiently, which would result in a reduction of losses. Nevertheless, the use of mineral fertilizers was classified as an inappropriate approach for this objective; reference was made both to the fact that the use of mineral N has been declining for several years and to the lack of fairness in terms of the polluter-pays principle. An instrument based on the mineral fertilizer price would address all agricultural entrepreneurs, whereas the experts agreed that only those actors with unacceptable nutrient losses should be sanctioned. The preliminary consensus reached within the working group was confirmed during the scenario workshop, establishing that the most appropriate technological starting point is an indicator closely aligned with the source of emissions, which is nutrient losses to the environment.

### Addressee

Given the consensus among experts that the technological starting point of a policy instrument should be closely aligned with the source of nutrient losses, it was agreed, both within the working group and during the plenary scenario workshop, that the primary addressees must be the emitters themselves, namely agricultural producers. Unlike mineral fertilizers, which are typically purchased and thus traceable through market transactions, a substantial share of organic fertilizers, such as manure, is generated and applied directly on-farm. Because these inputs are not necessarily traded or externally sourced, regulatory measures aimed at reducing nutrient losses must directly target the agricultural enterprise. Addressing the farm as the unit of intervention ensures that both mineral and organic nutrient flows are captured within the scope of the policy instrument.

### Regulatory Area

The identification of nutrient loss as the technological starting point and the agricultural entrepreneur as the primary addressee opens up a range of possible regulatory scales, ranging from national-level frameworks to field-specific interventions. However, the expert panel agreed that the farm level represents the most appropriate regulatory area for effectively addressing nutrient losses. This level of granularity ensures that the full spectrum of on-farm nutrient dynamics, including both mineral and organic sources, can be captured and managed. Broader control areas, such as regional or national averages of nutrient losses per hectare, were deemed unsuitable due to the high spatial variability of nutrient surpluses and the concentration of losses in specific farm types and regions. A farm-level regulatory approach thus allows for more targeted, equitable, and effective implementation of reduction objectives, aligning regulatory responsibility with the locus of environmental impact.

### Instrument

Building on the established consensus regarding the technological starting point, addressee, and regulatory area, the experts agreed that farm-gate nutrient balancing across all farm types represents an effective, outcome-oriented policy instrument for reducing N losses at the farm level (Eq. 1). They recommended that this approach should replace strongly action-oriented regulations of the Fertilizer Ordinance (DüV 2020).

**Equation 1: Farm-gate N balance**

1 $${Farm}-{gate}\,N\,{balance}\left[{kg}\frac{N}{{ha}}\right]=\frac{{Imported}\,N\,\left[{kg}N\right]-{Exported}\,N\,\left[{kg}N\right]}{{Utilised\; agricultural\; area}\,\left[{ha}\right]}$$

with:

$${Exported}\,N\,\left[{kg}\,N\right]=\sum \begin{array}{c}{Yield\; of\; marketed\; crops},{livestock},{animal\; products},\,\\ {mineral\; fertilizer},{organic\; fertilizer},\,\\ {seeds},{soil\; additives},{and\; plant\; material}\,\end{array}$$

$${Imported}\,N\,\left[{kg}\,N\right]=\sum \begin{array}{c}{Mineral\; fertilizer},{organic\; fertilizer},{fodder},{livestock},\\ {biological}N{fixation},\\ {seeds},{soil\; additives},{and\; plant\; material}\,\end{array}$$

In farm-gate balancing, nutrient flows are tracked using documentation such as invoices, delivery notes, and product declarations for N-containing inputs (e.g., mineral fertilizers, fodder), as well as standardized reference values (e.g., nutrient content of animal products, excretion coefficients). All N-containing products entering the farm from external sources are classified as “imported”, while those leaving the farm are considered “exported” (Löw et al., 2021).

According to the expert panel, the methodological design of a farm gate nutrient balance, which was added to German agri-environmental regulations in 2018 (StoffBilV 2017) and the applicable limit and loss values should be more ambitious and substantiated by scientific evidence. Also, the farm gate nutrient balance should be compulsory for all farm types. Currently, the gross farm gate balance threshold is an N surplus of 175 kg N/ha as a three-year mean. Alternatively, an individual farm maximum N surplus can be calculated. Additionally, loss factors are granted for organic fertilizers and roughage and must be added to the calculation (StoffBilV 2017; Löw et al., 2021).

Consensus was also reached on the need to harmonize the differing reference periods for N and P currently stipulated in legislation, in order to enhance consistency and coherence in nutrient management regulations. Participants noted that relevant literature is available on various regulatory approaches and their potential implications in the German context (Löw et al., 2021; Löw and Osterburg 2024). While the Delphi study did not define absolute threshold values for nutrient surpluses, there was agreement that standardized reference or surplus values should be grounded in scientific knowledge and integrated into the regulatory framework, accompanied by a reasonable transition period to allow farms to adapt.

Furthermore, the panel argued that the farm-gate nutrient balancing must be extended to include P application restrictions derived from the soil’s P supply prior fertilizer application at the field level. Establishing binding national P application limits depending on phosphorous content in the soil therefore is essential. The experts reached consensus that, on soils with a high P concentration in the root zone, fertilization should be restricted to match removal rates by the crop and complemented by erosion-protection measures, as already partially mandated in the Fertilizer Ordinance (DüV 2020). For heavily oversupplied soils, experts reached a consensus on imposing a fertilizer restriction of 20% below the extraction rate—a measure, the experts justified by the immobility of P in the soil, which necessitates a negative balance for long-term load reduction.

The implementation of comprehensive farm-gate nutrient balancing should be accompanied by enhanced control efficiency and monitoring intensity. To minimize administrative burden, the documentation and provision of operational data should leverage digital technologies, enabling streamlined data collection and reporting processes. The comprehensive monitoring of farm-gate balances is to be supplemented by on-site and accounting controls. There was a consensus that violations must be consistently sanctioned and circumventions systematically analyzed and then prevented. Furthermore, cross-checks must be possible in order to ascertain the reliability of the information provided by farmers. For instance, upstream suppliers (e.g., retailer providing mineral fertilizer) should be compelled to divulge their deliveries to farmers.

Furthermore, the expert group formulated the following consensus hypothesis: “Action-based regulatory requirements should only apply to farms that fail to comply with nutrient balance thresholds”. To ensure the effectiveness and fairness of this approach, experts emphasized the need for additional corrective measures specifically targeted at non-compliant operations. These measures could be either action-oriented interventions or market-based approaches, such as subsidizing desirable surpluses and/or a penalty on surpluses beyond defined thresholds. However, there was no majority within the expert panel on which instrument should be prioritized.

Additionally, the experts demanded the following research priorities to accompany policy implementation: site-specific fertilization and needs assessment, loss-reducing application (technology, acidification of manure, inhibitors) for organic fertilizers, recording nutrient levels of organic fertilizers, and breeding for higher N and P efficiencies in animal husbandry. The knowledge and processes gained then should be made available to farmers in order to enable rapid, broad implementation in agricultural practice. Therefore, research and producer-centric advice must be supported by appropriate public funding. It was determined that advice by extension service agencies on nutrient management constitutes a pivotal element in reducing the environmental impact of agriculture.

## Discussion

The application of a modified policy Delphi approach proved effective in addressing the research question, particularly in identifying starting points and design options for policy instruments that reflect the perspectives of key stakeholder groups and support the objectives of the Farm to Fork Strategy. While the study yielded valuable insights, further research is warranted, especially regarding the methodological refinement and practical implementation of the proposed measures. The findings presented here can serve as a foundation for subsequent studies aimed at defining plausible policy frameworks and assessing adaptation strategies at the farm level. The following discussion first examines the strengths and limitations of the modified Delphi methodology in relation to the research objectives. It then turns to a critical review of the substantive policy recommendations that emerged from the stakeholder process by the author.

### Modified Delphi Approach—Stakeholder Demand for Policy Development

In comparison to the classic Delphi method, a structured discussion process was chosen for the scenario design, aiming to combine iterative structured components of a Delphi study with the explorative components of focus group discussions (Linstone and Turoff 1975; Parker and Tritter 2006). Unlike the classic Delphi, this design did not rank stakeholder statements or quantify the importance of divergent viewpoints. Instead, it prioritized open dialog and deliberation from the outset, fostering a discursive environment that encouraged the articulation of diverse arguments and underlying rationales. This orientation aligns with the recommendations of Reed (2008), who emphasizes the value of participatory approaches that grant stakeholders genuine influence over outcomes. The opportunity for key stakeholders to gradually deconstruct the policy issue into its individual dimensions, jointly discuss implementation options, and negotiate common ground was also greatly appreciated by the participating experts.

A key feature of the approach was the structured use of policy design parameters developed by Scheele et al. (1992), which provided a common framework for analyzing and comparing alternative instruments. These parameters—technological starting point, regulatory area, addressee, costs, and instrument type – helped focus the discussions and were successfully applied in previous scenario studies, including on pesticide reduction (Dehler 2023). Their use in this study enabled cumulative learning, as participants revisited and refined their positions across thematic sessions. This iterative, criteria-based structure has been similarly endorsed in recent governance and policy design literature (Howlett et al., 2015; Jordan and Turnpenny 2015).

The high level of consensus observed among participants may raise concerns about potential selection bias. While it is acknowledged that not all participants were able to attend every workshop, the sampling strategy was deliberately designed to emphasize institutional relevance and balanced stakeholder group representation across the agriculture, environmental protection, and academia, rather than demographic diversity. Experts were chosen based on their affiliation with influential organizations and their ability to represent the broader perspectives of their sectors. To mitigate potential bias, the process ensured that all stakeholder groups were consistently represented across the various stages of the Delphi process.

Importantly, the consensus achieved emerged through intensive, structured discussions in which participants critically examined trade-offs, advantages, and drawbacks of different policy approaches. The iterative nature of the process allowed participants to engage in continuous reflection, revisit earlier discussions, and refine their positions, thereby strengthening the robustness and validity of the resulting policy recommendations.

Nevertheless, the possibility of groupthink, the tendency for group dynamics to suppress dissenting viewpoints in pursuit of consensus, cannot be fully excluded. While measures such as neutral moderation, structured thematic blocks, and reflection phases were implemented to foster open dialog and mitigate convergence bias, it remains a potential limitation inherent to deliberative processes. Future studies may consider incorporating anonymous feedback rounds or parallel individual assessments to further reduce this risk, as done by Ehlers et al. (2022).

In accordance with Turoff’s (1970) guidance, participants were provided with a preparatory dossier summarizing key legislation, scientific literature, and policy design methodology prior to participation. Providing structured background materials has been shown to promote more active engagement and informed discussion (Richards. et al., 2004; Welp et al., 2006; Reed et al., 2009; Wright and Cairns 2011). Furthermore, flexibility in scheduling and the use of online formats were essential in accommodating participants’ professional commitments. Workshops were planned at least six weeks advance, and the virtual setting facilitated consistent involvement and high levels of engagement throughout the iterative process.

### Outcomes of Stakeholder-Driven Policy Development

Stakeholders engaged in the Delphi process expressed a strong preference for an ambitious farm-gate nutrient balancing as a outcome-oriented approach to nutrient management, supplemented by targeted measures for farms that fail to meet prescribed balance thresholds. This shift was considered essential to ensure that regulatory efforts yield measurable environmental improvements compared to the current situation. The findings reflect a growing demand for fertilizer policies that are more closely aligned with the operational diversity and practical realities of agricultural systems, a perspective that is well supported by recent literature (Iversen et al., 2024; Löw and Osterburg 2024).

As outlined in the results section, experts emphasized that the current regulatory threshold should be made more ambitious and scientifically substantiated. In the discussion, some participants raised concerns about specific calculation elements for the farm-gate N balance, such as the treatment of losses from intensive grassland systems. These concerns underscore the need for further scientific evidence, improved transparency, and indicators, that are robust enough to serve as a credible basis for policy enforcement and farm-level decision-making.

A harmonized methodology for defining P application limits is essential, particularly one that accounts for soil P content and corresponding balance surpluses. Currently, regulatory standards vary considerably across federal states (The Federal Government, 2021). In this context, the expert panel’s consensus, advocating for fertilizer application to be restricted to 20% below crop P removal rates on heavily oversupplied soils, represents a significant departure from existing legislation. This recommendation underscores the need for more ambitious and scientifically grounded regulation.

Various methodological approaches for both N and P balancing have been proposed in the literature, and the validity and reliability of farm-gate nutrient balancing as a policy tool have been well documented (Klages et al., 2017; Taube et al., 2020; Löw et al., 2021; The Federal Government., 2021; Löw and Osterburg 2024). Existing literature, along with identified regulatory vulnerabilities, such as inconsistent P thresholds or current surplus allowances, provide a valuable foundation for refining instrument design and guiding future research.

This also applies to the comparatively high administrative and control costs associated with farm-gate nutrient balancing, as opposed to a mineral fertilizer tax, a topic that was a focal point of discussion. While experts acknowledged the lower enforcement costs of a fertilizer tax, they emphasized its limited effectiveness in addressing nutrient surpluses at the farm level. In contrast, farm-gate balancing directly targets farm-specific nutrient performance, sanctioning only those exceeding acceptable thresholds. Löw et al. (2021) argue that digital, receipt-based documentation of nutrient flows—from farms to the broader value chain—could significantly enhance data acquisition, reliability, and overall transparency. The integration of artificial intelligence (AI)-supported analyses would allow for the timely identification of anomalies and inefficiencies, ultimately improving the quality of information available to farmers, enhancing advisory services, and increasing the operational efficiency of control authorities. These statements are in line with the stakeholder demand strongly emphasizing the desirability of making use of the potential of digitalization to reduce administrative expenses and foster acceptance of nutrient balancing measures.

An important issue that remained unresolved during the Delphi process concerns the integration of farm-gate nutrient balancing with market-based instruments for farms that fail to comply with defined surplus thresholds. While stakeholders broadly supported the principle of differentiated regulation based on performance, there was no consensus on how such instruments (such as levies, tradable permits, or subsidies) should be operationalized in practice. This gap highlights a critical area for further exploration. Fortunately, a substantial body of literature exists that examines the design, implementation challenges, and potential impacts of economic instruments in nutrient management, offering valuable insights for future policy development (Isermeyer and Schleef 1994; Grethe et al., 2021; Martínez-Dalmau et al., 2021; Garske et al., 2025).

Further, the adoption of innovative technologies such as site-specific application or acidification of manure in general offers promising avenues to optimize nutrient management, ensuring compliance with nutrient limits and reducing surpluses more efficiently. In this context, future research should concentrate on the evaluation of the specific contributions of various technologies and innovative management strategies at regional levels, with the objective of fully realizing potential efficiency gains.

## Conclusions

This study brought together experts from agriculture, environmental protection, and academia to assess how fertilizer policy in Germany can be developed further. Participants emphasized that regulatory efforts should focus on measurable outcomes rather than prescriptive practices. Farms exceeding scientifically defined nutrient surplus thresholds should be subject to targeted requirements or incentives, while those operating within acceptable limits should benefit from reduced regulatory burdens. However, no consensus was reached on what constitutes acceptable balance values or threshold levels for nutrient surpluses. Defining these thresholds, based on scientific evidence, remains a critical task for future research and policy development.

The modified Delphi methodology applied in this study proved effective in fostering deliberation and consensus across diverse stakeholder groups. The broad support for outcome-based measures suggests their potential applicability in other policy contexts and geographic settings. Nonetheless, further methodological refinement is needed to enhance the robustness and transferability of this approach. Future research should focus on improving stakeholder diversity, increasing transparency in consensus-building processes, and testing proposed instruments through pilot projects to assess their feasibility, effectiveness, and broader policy implications.

## Supplementary Information

Appendix A_Supplementary material

## Data Availability

The qualitative data generated and analyzed during this study, including interview and workshop transcripts, are not publicly available due to confidentiality and anonymity agreements with participating experts. Sharing these materials would compromise the privacy of individuals involved. Summarized insights and methodological details are provided within the manuscript.
