# Supplementary material for: Stakeholder Perspectives on Fertilizer Policy in Germany: Findings from a Modified Delphi Study
Source: Environ Manage. 2025 Sep 27;75(12):3220–33. doi: 10.1007/s00267-025-02266-3 (PMC12575518; doi:10.1007/s00267-025-02266-3)
Supplement: Supplementary file 1 — Appendix A_Supplementary material [file 267_2025_2266_MOESM1_ESM.docx]

Supplementary material for article on „Stakeholder Perspectives on Fertilizer Policy in Germany: Findings from a Modified Delphi Study” in Environmental Management

Jannik Aaron Dresemann*

Thünen Institute of Farm Economics, Bundesallee 63, 38116 Braunschweig, Germany

*E-mail address: [jannik.dresemann@thuenen.de](mailto:jannik.dresemann@thuenen.de); ORCiD: https://orcid.org/0009-0002-9097-277X

Content

[1 Workgroup fertilization: 1^st^ meeting questionnaire and agenda 2](#_Toc205620775)

[2 Workgroup fertilization: 2^nd^ meeting questionnaire and agenda 4](#_Toc205620776)

[3 Scenario workshop: Agenda 5](#_Toc205620777)

Note: The following questionnaires and agendas were originally prepared in German and have been translated into English for inclusion as supplementary material. Minor editorial adjustments were made for clarity without altering the content.

# Workgroup fertilization: 1^st^ meeting questionnaire and agenda

Objectives and approach of the workgroup fertilization

Overall objective

The working group will develop a policy scenario for an economic impact assessment of the Farm2Fork fertilizer strategy. The aim is to define how the 50% reduction of nutrient losses should be implemented politically in order to determine individual farm impacts and adaptation options. To this end, stakeholders from agriculture, environmental protection and science should exchange their positions and, if possible, reach a consensus on which national policy instruments should be used to achieve the targets.

Exogenous framework assumptions

- In principle, existing regulations should be built upon and the policy architecture should be complemented in a meaningful way.
- The national balance serves as a reference for the development of nitrogen surpluses. For a 50% reduction, the total balance must not exceed 50 kg N/ha.

Overarching research questions

- What new or modified policy instruments, or combinations thereof, are considered appropriate to motivate farmers to reorganize their production in such a way that the objectives of the Farm2Fork strategy in the area of plant protection and fertilization are achieved with limited public resources?
- How should policy instruments that are considered appropriate be designed?
- How to minimize trade-offs, in particular between sustainability aspects and economic interests?

Specific questions in the field of fertilization

- How do you assess policy instruments that are currently being introduced or are in the process of being adapted (e.g. the fertilizer ordinance or the material flow balance ordinance) in relation to the Farm2Fork strategy's goal of reducing nutrient losses for a) nitrogen and b) phosphate? Why is this the case?
- How could current policy instruments be further developed to most efficiently capture and reduce a) nitrogen and b) phosphorus losses in line with the target? Where should they start (e.g. management practices, crop rotations, nutrient balancing) and how should they be designed?
- Should new policy instruments be introduced to achieve reduction targets more efficiently (e.g. nitrogen tax, regional livestock limits)? At what spatial level (national, regional, individual farm, area-specific) should regulation take place, where should policy instruments start (e.g. product, management practices, advisory) and how could they be designed?

**First meeting workgroup fertilization**

Meeting objectives

1. Consensus and dissent are developed on whether policy instruments currently in use or about to be adapted are suitable for reducing nutrient losses by 50% in line with requirements.
2. If there is consensus that currently applied policy instruments will not lead to sufficient loss reductions, consensus should be reached as far as possible on a) how current policy instruments (e.g. the Fertilizer Ordinance or the Nutrient Flow Balance Ordinance) should be expanded and/or b) which new policy approaches should be pursued in order to achieve a further reduction in nutrient losses.

The motives that led to consensus or consensus over dissent in the individual objectives must be documented.

Suggestions for the agenda

1. Introduction, mutual introductions, objectives, procedures, and framework for the discussion session. Ask about the possibility of recording the meeting.
2. Summary of the assumptions made beforehand from the concept and the dossier, which form the framework for the discussion.
3. Each participant answers the question in an elevator pitch style:

What is your vision for future fertilizer policy to effectively reduce nutrient losses at the national level? (2min per participant)

1. Brainstorming in two small groups on the main challenges of the current fertilizer policy to efficiently achieve the nutrient loss reduction targets of the Farm2Fork strategy. The following questions should be answered by both groups:
   1. How do you assess the policy instruments currently in place or in the process of being adapted (e.g. the fertilizer ordinance or the material flow balance ordinance) in relation to the reduction of nutrient losses for a) nitrogen and b) phosphate targeted by the Farm2Fork strategy? Why?
   2. In your opinion, can the reduction targets for nitrogen be achieved more efficiently through a) the further development of existing policy instruments or b) the introduction of new policy instruments? Why?
   3. At what spatial level (national, regional, individual farm, area specific) should regulation take place, where should instruments start (e.g. product, management practices, advice) and how could they be designed?

The aim is to group views according to consensus and dissent. Topics on which consensus cannot be reached during the brainstorming session, or which seem particularly relevant to individual experts, will be collected in the "ideas parking lot" and followed up separately.

1. Presentation, consolidation and discussion of group results.
2. Summary, feedback and outlook.

Follow-up

- The study's initiator will draft a protocol, analyze the meeting's results, and develop an initial concept for possible instruments. This draft will be distributed to all participants before the next working group meeting, serving as a new basis for discussion.
- Once the topic of nitrogen has been completed, the question of objectives and procedures for phosphate losses will be discussed.

# Workgroup fertilization: 2^nd^ meeting questionnaire and agenda

Objectives and approach of the workgroup fertilization

**Second meeting workgroup fertilization**

Meeting objectives

1. Consensus should be reached as far as possible on which of the design alternatives (base value, differentiation of losses, permissible balance value) of material flow balancing is preferred with regard to a) nitrogen and b) phosphorus and how the selected option should be further developed.
2. As far as possible, a consensus should be reached on whether further measures (e.g. the promotion of technology or low-emission management practices) appear necessary.

The motives for the respective decisions must be documented.

Suggestions for the agenda

1. Introduction, main results of the first discussion round, objectives, procedure and framework conditions for the discussion round.
2. Evaluation of the design alternatives for material flow balancing with regard to a) nitrogen and b) phosphorus with the aim of ideally agreeing on one alternative as the preferred option. The starting point is the dossier sent out in advance.
3. Brainstorming on possible further measures in fertilizer policy to identify accompanying options that make it possible to reduce the balance values efficiently and in a targeted manner.
4. The aim is to group the views according to consensus and dissent. Topics on which no agreement is reached or which appear particularly relevant to individual experts are followed up separately.
5. Summary, feedback and outlook.

Follow-up

- The initiator of the study will draft a protocol of the results, further elaborate the concept for the areas of material flow balancing and further measures, and summarize possible design alternatives. This elaboration will be distributed to all participants before the next meeting of the working group, serving as a new basis for discussion.
- If the group determines that further meetings are not necessary to elaborate on the fertilization area, the initiator will prepare the results for discussion at the concluding workshop.

# Scenario workshop: Agenda

Objectives and approach of the plenary workshop

Objectives

1. All experts should be given the opportunity to comment on the results of the working group.
2. The individual core components of the working group results should be refined, and the expert group should vote on the scenarios developed for the areas of fertilization and plant protection.

The reasons behind the consensus or consensus over dissent in the individual objectives must be documented.

Suggestions for the agenda

1. Introduction, review, objectives, procedure and framework conditions for the workshop.
2. Summary of the discussion process and the results from the working group on plant protection. Key components are presented in turn and then discussed based on guiding questions (e.g., regarding policy instruments or the Dutch cooperative model). The starting point is the dossier that was sent out in advance.
3. Summary of the discussion process and the results from the working group on fertilization. Key components are presented in turn and then discussed based on guiding questions (e.g., regarding the regulation of organic nitrogen input or the promotion of low nutrient balance surpluses). The starting point is the dossier that was sent out in advance.
4. Summary, feedback and outlook.

The objective of the in-depth discussions is to document the perspectives on sometimes crucial decisions. The arguments are pursued independently, and the subjects where no consensus is achieved are documented as dissenting opinions.

Follow-up

- The initiator of the study will develop the concept for a policy scenario in the area of fertilization, including both the results of the working group and those of the final workshop. The areas of consensus will be adopted, and the various design alternatives will be presented for aspects on which there was consensus on dissent. The draft will be distributed to all participants, as will the preparatory dossiers that were made available to the working groups during the discussion rounds.
- Upon approval by the participants, the findings from the process will be translated into a scientific publication. Additionally, the findings will be offered to be published jointly in an agricultural or environmental magazine and/or presented at the respective experts' institutions.
